# Tailoring biocompatibility of composite scaffolds of collagen/guar gum with metal–organic frameworks

**DOI:** 10.1039/d1ra08824f

**Published:** 2022-01-27

**Authors:** Martín Caldera-Villalobos, Denis A. Cabrera-Munguía, Juan J. Becerra-Rodríguez, Jesús A. Claudio-Rizo

**Affiliations:** Facultad de Ciencias Químicas, Universidad Autónoma de Coahuila Ing. Cárdenas Valdez S/N Saltillo Coahuila México mcalderafcq@uadec.edu.mx jclaudio@uadec.edu.mx; Universidad Politécnica de Pénjamo Carretera Irapuato – La Piedad Km 44 Pénjamo 36921 Guanajuato México

## Abstract

Metal–organic frameworks (MOFs) are microporous materials with high potential for biomedical applications. They are useful as drug delivery systems, antibacterials, and biosensors. Recently, composite materials comprised of polymer matrixes and MOFs have gained relevance in the biomedical field due to their high potential as materials to accelerate wound healing. In this work, we studied the potential applications of composite hydrogels containing MgMOF74, CaMOF74, and Zn(Atz)(Py). The composite hydrogels are biodegradable, being completely degraded after 15 days by the action of collagenase and papain. The composites showed high biocompatibility reaching cell viabilities up to 165.3 ± 8.6% and 112.3 ± 12.8% for porcine fibroblasts and human monocytes, respectively. The composites did not show hemolytic character and they showed antibacterial activity against *Escherichia coli* reaching up to 84 ± 5% of inhibition compared with amoxicillin (20 ppm). Further, the immunological assays revealed that the composites produce a favorable cell signaling stimulating the secretion of the TGF-β and MCP-1 cytokines and maintaining the secretion of TNF-α in normal levels. Finally, the composites showed potential to be used as controlled drug delivery systems reaching a release efficiency of 30.5 ± 2.5% for ketorolac. Finally, results revealed that ColGG-Zn(Atz)(Py) was the best formulation evaluated.

## Abstract 2

MOF Zn(Atz)(Py) tailored the biocompatibility of collagen/guar gum hydrogels stimulating the cell metabolism and the secretion of TGF-β and MCP-1. Further, Zn(Atz)(Py) increased the antibacterial activity and improved the drug release performance.

## Graphical abstract

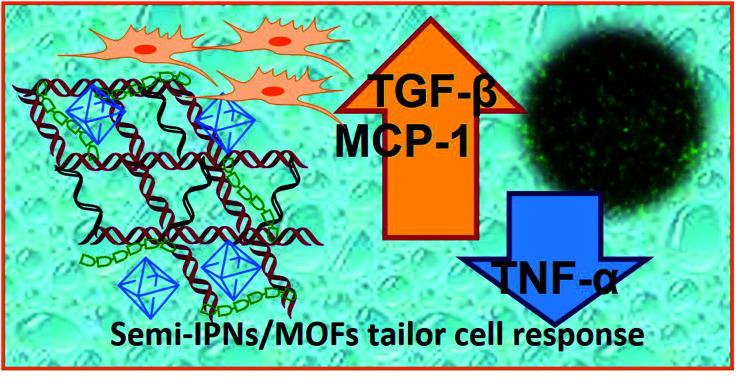

## Introduction

MOFs are polymeric materials consisting of metal ions linked together by organic bridging ligands.^1^ This class of mesoporous materials has gained significant scientific attention due to their high surface areas, uniform porosity over various length scales, high-volume storage capability, shape selectivity, enhanced mass transport, and the ability for diffusion.^2^ The ultrahigh porosity, the high internal surface area, and the pore opening make MOFs suitable materials for the inclusion and capture of small molecules. Thus, MOFs are relevant materials for the selective separation of ions,^3^ removal of textile dyes^4^ and heavy metals,^5,6^ catalysis of several organic reactions,^7,8^ solar cells^9^ and other optoelectronic devices,^10^ and biomedical applications.^11–16^

The applications of MOFs in the biomedical field include drug delivery systems, delivery of biomolecules, intracellular trafficking, antibacterial, cancer therapy, photodynamic therapy, gas storage, biosensors, and diagnosis.^11^ Further, MOFs may be found applied as contrast agents for magnetic resonance imaging.^12^ However, the biodegradation of MOFs is important to avoid bioaccumulation in living organisms. The design of biodegradable MOFs requires choosing metals and ligands with minimal toxicities. Some metal ions for this purpose are Fe^2+^, Fe^3+^, Ca^2+^, Zr^2+^, Co^2+^, Mn^2+^, Mg^2+^ and Zn^2+^ which are included in the composition of the human body as microelements.^13^ Rare earth metal–organic frameworks are promising materials for biomedical applications such fluorescence/magnetic resonance imaging, drug delivery, and biomedical sensing.^14^ The toxicity of MOFs depends strongly on the nature of the metal atoms and the constitutive organic linkers.^15^ The careful selection of the building blocks and their combination with biocompatible metal nodes is the fundamental step to building bioMOFs.^16^ Several biological ligands are available for the design and synthesis of MOFs for biomedical applications. They include amino acids, peptides, nucleobases, proteins, porphyrins, saccharides, and vitamins.^17,18^

There are several examples of relevant MOFs in the biomedical field. Zn MOFs are considered safe for theragnostic applications. Zn is a relevant element to important biological functions and Zn-based MOFs show low toxicity.^19^ The copper-based MOF named HKUST is degraded rapidly in a protein solution releasing Cu^2+^ ions which stimulate angiogenesis.^20^ MOFs based on gallic acid show potential as antioxidant carriers and anticancer agents.^21^ Porphyrin-based MOFs have excellent properties to combine two or more therapy modalities for tumor therapy, such as photodynamic therapy and drug delivery.^22^ Bimetallic MOFs containing Ti and Zr showed excellent performance in wound dressings due to their photocatalytic activity, generating reactive oxygen species which eliminate multidrug-resistant bacteria.^23^ Additionally, the surface engineering of MOFs with unique functional materials can overcome issues associated with conventional wound healing methods.^24^

As mentioned afore, MOFs are excellent host materials due to their high and tunable porosity. Thus, they avoid the leaching of the loaded substrates and provide a protective environment against external adverse effects.^25^ ZIF-67 was used for the controlled release of a pro-angiogenic drug for accelerating chronic wound healing.^26^ Other drugs encapsulated in MOFs include ibuprofen, doxorubicin, busulfan, 5-fluorouracil, and caffeine reaching up to a rate release of 50%.^27^

Further, MOFs are interesting materials in the biomedical field due to the antibacterial activity provided by the metal ions or organic linkers. For example, the antibacterial activity of ZIF-8 is attributed to the Zn^2+^ release from the MOF surface destroying the structure of the cell membrane leading to bacterial cell death.^28,29^ The antibacterial activity was observed in other MOFs such as HKUST,^30,31^ Cu-MOFs,^32^ and Ag-MOF.^33^

Frequently, the biomedical applications of MOFs require the design and formulation of composite materials where MOFs are the disperse phase. Several approaches are available for the synthesis of composites containing MOFs: non-covalent attachment, covalent attachment, polymer coordination to metal ions, and MOF encapsulation in polymers.^34^ Composite materials containing MOFs have shown great potential for therapeutic applications. Niacin-based MOFs encapsulated in alginate microcapsules release Cu^2+^ and Zn^2+^ ions and they act as auxiliary for chronic wound healing.^35^ Also, the release of Cu^2+^ ions from HKUST encapsulated in a thermoresponsive hydrogel accelerates wound healing in diabetic mice by a cooperative effect between the antibacterial activity of copper and the mitigation of cytotoxic effects by the hydrogel.^36^ The synergy of materials for protecting against bacterial infections was observed in poly(vinyl alcohol)/UiO-66-NH_2_ composites which showed higher performance than commercially available gauzes used for the wound healing process.^37^ Further, the synergistic antibacterial activity between polymers and MOFs has been observed in polyacrylonitrile/gelatin nanofibers coated with ZIF-8 loaded with gentamicin to accelerate the wound repair.^38^

**Chart 1:**
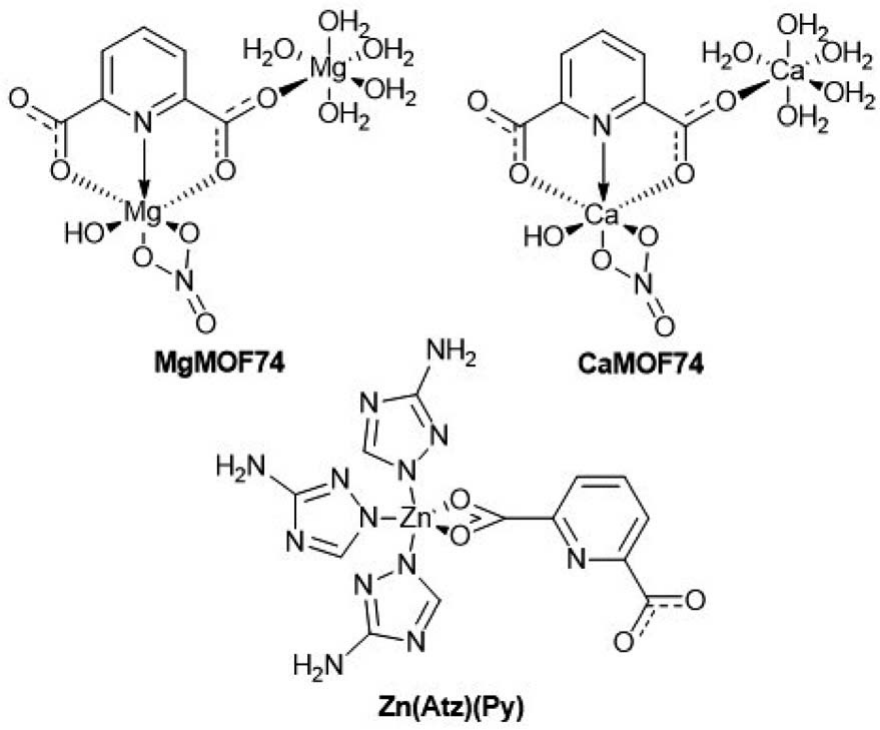
Chemical structures of MOFs.

Recently, our group studied the biomedical potential of a new MOF based on Al^3+^ and bis(hydroxyethyl)terephthalate founding a non-cytotoxic character and great potential for controlled drug release.^39^ In addition, also recently it was reported the generation of hydrogel biomatrices based on semi-interpenetrated networks of collagen-polyurethane-polysaccharides with potential properties for biomedicine such as mechanical and degradation.^40^ Specifically, in the field of scaffolds for wound healing these matrices require properties such as accelerated gelation in the wound bed, antibacterial capacity, controlled release of drugs and modulation of secretion of important cytokines in healing by cells of the immune system, these characteristics could be tailored in biomatrices by including MOFs. The present work aimed to study the biomedical application of composite hydrogels containing three MOFs named MgMOF74, CaMOF74, and Zn(Atz)(Py) which contains Mg^2+^, Ca^2+^, and Zn^2+^ ions able to tailoring the cell response (Chart 1). MOFs were synthesized from heterocyclic ligands, and dispersed in a hydrogel matrix comprised of collagen, polyurethane and guar gum forming composite scaffolds. The biological activity of composite hydrogels was studied through cell viability, cell proliferation, quantification of cytokines related to wound healing in human monocytes by enzyme-linked immunosorbent assay (ELISA), and bacterial inhibition. Also, the ketorolac release from the composite hydrogels was studied. The results showed that the studied composites have great potential for biomedical applications such as wound healing.

## Results and discussions

### Characterization of MOFs

**Fig. 1:**
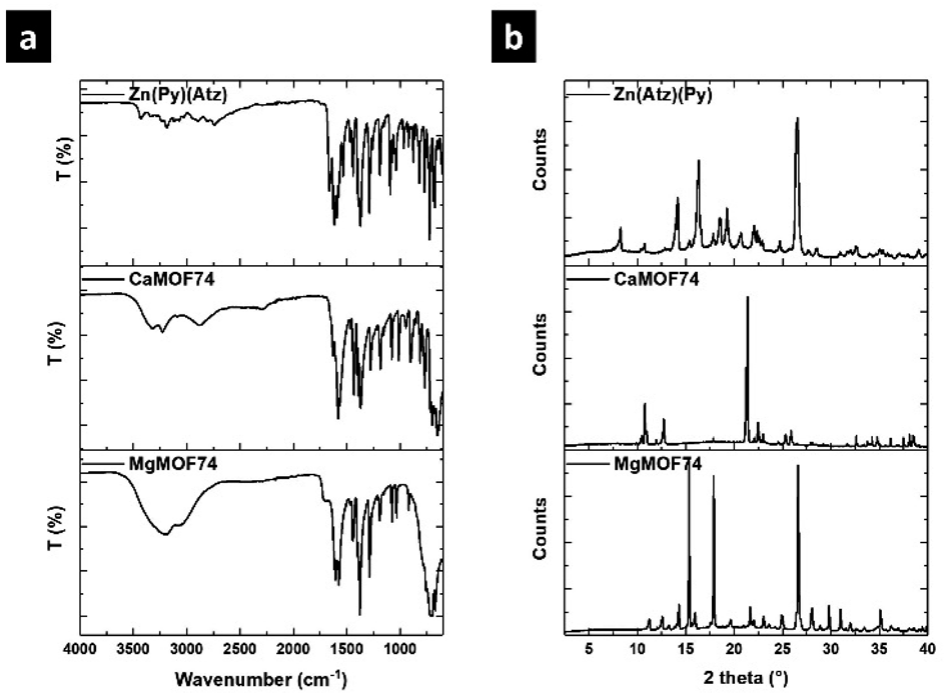
(a) FTIR spectra, and (b) XRD patterns of MOFs.

MgMOF74, CaMOF74, and Zn(Atz)(Py) were synthesized under hydrothermal conditions and they were characterized by FTIR, XRD, and SEM. Fig. 1a shows FTIR spectra of MOFs. The FTIR spectrum of MgMOF74 shows two absorption bands at 1611 and 1577 cm^−1^ attributed to the stretching vibrations of the pyridine ring from the ligand H_2_Py. The band due to the νC=O vibration from the carboxylate groups was observed at 1716 cm^−1^. Further, the bands observed at 1289, 1196, 1036, and 1018 cm^−1^ were assigned to the νC–O vibration from carboxylate groups. The vibration band of the Mg–O coordination bond is appreciated at 660 cm^−1^. Finally, the band at 3200 cm^−1^ was attributed to the νO–H vibration of water molecules adsorbed in the MOF. The spectrum of CaMOF74 is like the spectrum of MgMOF74 because both were synthesized from the H_2_Py ligand. The stretching vibrations from the pyridine ring were observed at 1626 and 1585 cm^−1^ respectively. The asymmetric and symmetric N–O vibrations from the nitrate ion were observed at 1464 and 1367 cm^−1^ respectively. The bands due to the νC–O vibration were observed at 1275, 1188, 1152, 1078 and 1011 cm^−1^ respectively. The vibration band of the Ca–O coordination bond is appreciated at 740 cm^−1^. Finally, the band due to the νO–H vibration from the adsorbed water was observed at 3209 cm^−1^. The FTIR spectrum of Zn(Atz)(Py) shows three absorption bands at 1662, 1624, and 1613 cm^−1^ due to the νC=N vibration. The first and second bands are related to the triazole ring from the HAtz ligand while the third is due to the pyridine ring from the H_2_Py ligand. The bands observed at 1291, 1259, and 1190 cm^−1^ were assigned to the νC–O vibration from carboxylate. The vibration band of the Zn–O coordination bond is appreciated at 690 cm^−1^. Finally, the band at 3423 cm^−1^ was attributed to the asymmetric stretching vibration of the free amino groups of the ligand HAtz.

The crystallinity of the synthesized MOFs was analyzed by XRD, and the respective patterns are shown in Fig. 1b. The three patters show strong diffraction peaks showing the high crystallinity of MOFs. The main diffraction peaks were observed at 15.3, 17.9, and 26.6° for MgMOF74, while the main peaks for CaMOF74 were observed at 10.8, 12.7, 21.2, and 21.3°. Finally, the main diffraction peaks of Zn(Atz)(Py) were observed at 14.2, 16.3, and 26.5°. The diffraction patterns for MgMOF74 and Zn(Atz)(Py) are associated with a rod crystal arrangement, while the diffraction pattern for CaMOF74 is related to a prismatic crystal arrangement.

**Fig. 2:**
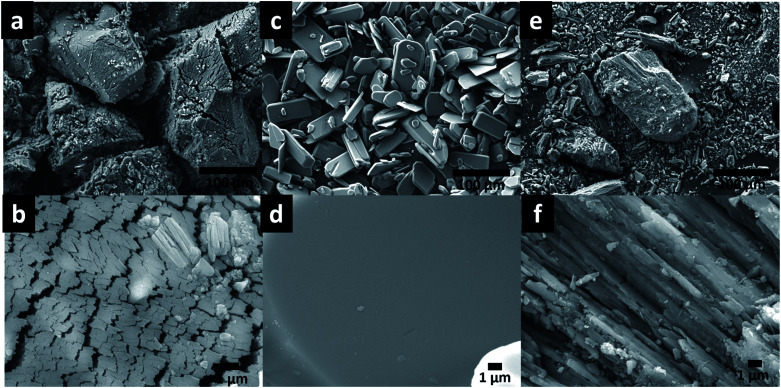
SEM images of (a and b) MgMOF74, (c and d) CaMOF74, and (e and f) Zn(Atz)(Py).

The morphology of MOFs was observed by SEM and the acquired images are shown in Fig. 2. MgMOF74 and Zn(Atz)(Py) are formed by particles with and irregular shape (Fig. 2a and e), these particles are arranged to generate flat surfaces based on microrods (Fig. 2b and f); while the particles of CaMOFF4 have a prism-like shape (Fig. 2c), these particles associate to generate smooth surfaces (Fig. 2d).

### Formation of composite hydrogels

**Scheme 1:**
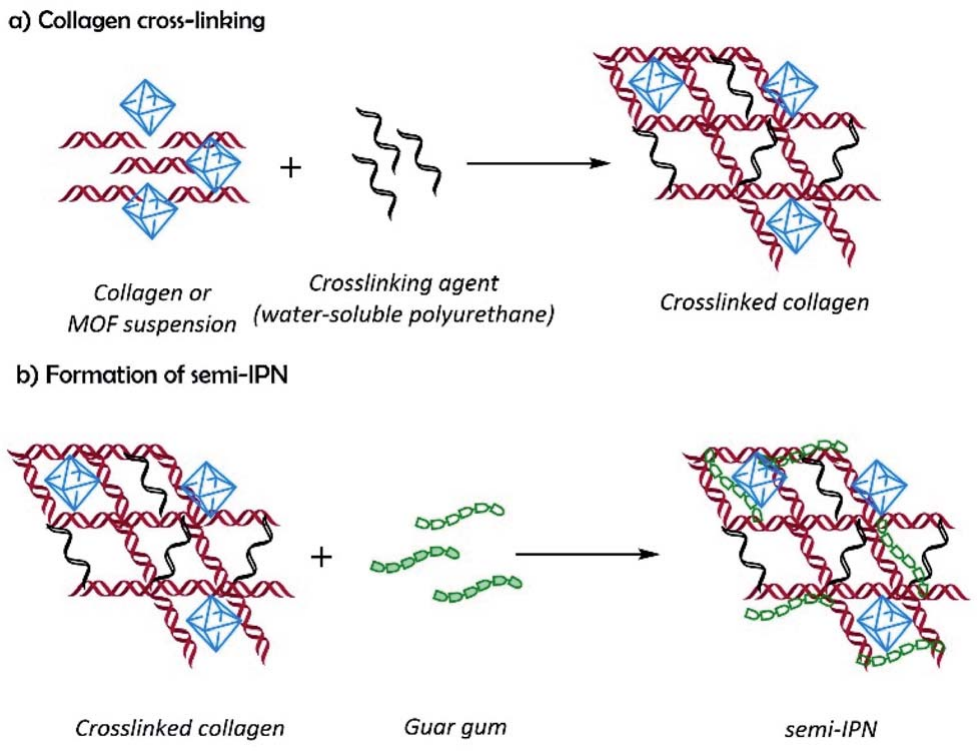
Schematic representation for the preparation of composite hydrogels: (a) crosslinking of the polymer matrix, and (b) semi-interpenetration.

**Fig. 3:**
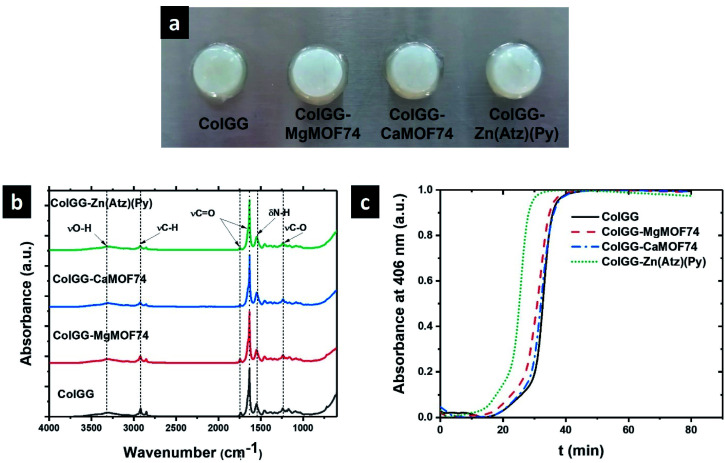
(a) Photography of composite hydrogels, (b) FTIR spectra, and (c) gelling kinetic curves.

Composite hydrogels were obtained by the reaction of collagen with the polyurethane at 37 °C in the presence of guar gum, and MOFs yielding semi-IPN matrixes with dispersed MOF particles, where collagen and polyurethane are covalently bonded by urea groups; and guar gum and MOFs are physically associated with the matrix by hydrogen bonding (Scheme 1). Composite hydrogels acquired the mold shape (Fig. 3a), and the formation of semi-IPNs was confirmed by FTIR (Fig. 3b). The spectra show the typical absorption bands observed for collagen: amide I (1654, 1631 cm^−1^), amide II (1550 cm^−1^), and amide III (1128 cm^−1^). The bands related to guar gum were observed at 3394 cm^−1^ (νO–H), 2922 cm^−1^ (symmetrical stretching of methylene groups), and 1032 cm^−1^ (νC–O) which are common for polysaccharides. The weak band at 1735 cm^−1^ was assigned to the νC=O vibration from urea groups. This band is evidence of the formation of the semi-IPN network by the crosslinking reaction between amino groups from collagen and isocyanate groups from polyurethane without involving the hydroxyl groups from guar gum. The interaction between MOFs and the polymers matrix was observed by the shifting of the νC–O band from 1172 to 1160 cm^−1^, and the absence of the band at 1096 cm^−1^ (νC–O). These changes suggested an interaction between the polymer matrix and the free carboxyl groups of MOFs by secondary bonds.

The maximum water uptake capacity, crosslinking degree, and storage moduli, were previously reported, and data are presented in Table 1.^41^ Interestingly, as reported, the metal ion in the MOF allows tailoring the physicochemical properties of the composite matrix. The MgMOF74 and CaMOF74 improve the crosslinking and the mechanics of the matrix, Zn(Atz)(Py) the swelling capacity; the three MOFs enhance the stability to thermal degradation. The regulation of physicochemical properties is a fundamental aspect for success in biomedical applications.^41^

**Table 1:** Physicochemical properties of composite hydrogels^41^

Hydrogel	Maximum water uptake capacity (%)	Crosslinking degree (%)	*G*′^a^ (Pa)
ColGG	2047 ± 47	47.4 ± 3.3	19.9
ColGG-MgMOF74	2185 ± 133	52.0 ± 4.4	87.9
ColGG-CaMOF74	2171 ± 117	50.5 ± 2.7	43.5
ColGG-Zn(Atz)(Py)	2301 ± 287	46.5 ± 4.3	11.2

a Measured at 1 Hz.

The *in situ* gelation ability is highly desirable for wound healing applications. For this, the formation of polymeric matrixes was studied by the turbidimetric analysis showing the gelling kinetics (Fig. 3c). Sigmoidal curves were obtained featuring the stages of nucleation (phase lag), growth, and plateau. Table 2 contains the lag time (*t*_lag_), polymerization rate during the growth phase (*S*), and the average polymerization time (*t*_1/2_) of the hydrogel matrixes formed by collagen, polyurethane, and guar gum. The lowest *t*_1/2_ corresponds to the ColGG-Zn(Atz)(Py) composite whereas the highest was found for ColGG. Similarly, the lowest *t*_lag_ is found for ColGG-Zn(Atz)(Py) and the highest corresponds to ColGG and the ColGG-CaMOF74 composite. Finally, the highest *S* corresponds to ColGG-Zn(Atz)(Py) and the lowest is found for ColGG-CaMOF74.

**Table 2:** Kinetic parameters for the formation of composite hydrogels

	ColGG	ColGG-MgMOF74	Col-GG-CaMOF74	ColGG-Zn(Atz)(Py)
*t*_lag_ (min)	16 ± 0.8	13 ± 2.4	16 ± 0.4	7 ± 1.3
*t*_1/2_ (min)	29 ± 1.0	25 ± 1.5	26 ± 1.2	21 ± 0.6
*S* (min^−1^)	0.1490 ± 0.03	0.0973 ± 0.03	0.0834 ± 0.06	0.1541 ± 0.02

The reduction of the polymerization time of the composites could be due to a catalytic effect produced by the MOFs. It is well known that the polymerization reactions of isocyanate monomers are catalyzed by bases, typically tertiary amines such as 1,4-diazabicyclo[2.2.2]octane (DABCO).^42^ Therefore, MOFs may catalyze the gelling reaction due to the presence of heterocyclic ligands with basic character. Further, the metal centers of MOFs are Lewis acids and they can coordinate to the oxygen atom of the NCO group and activate the electrophilic nature of the carbon atom.^43^ The synergy of basic heterocyclic ligands and the acid sites of metal ions could make MOFs efficient catalysts for collagen crosslinking accelerating the formation of matrices in the hydrogel state. The best catalytic effect was observed using Zn(Atz)(Py) forming the crosslinked polymeric matrix after 7 ± 1.3 min at body temperature.

**Fig. 4:**
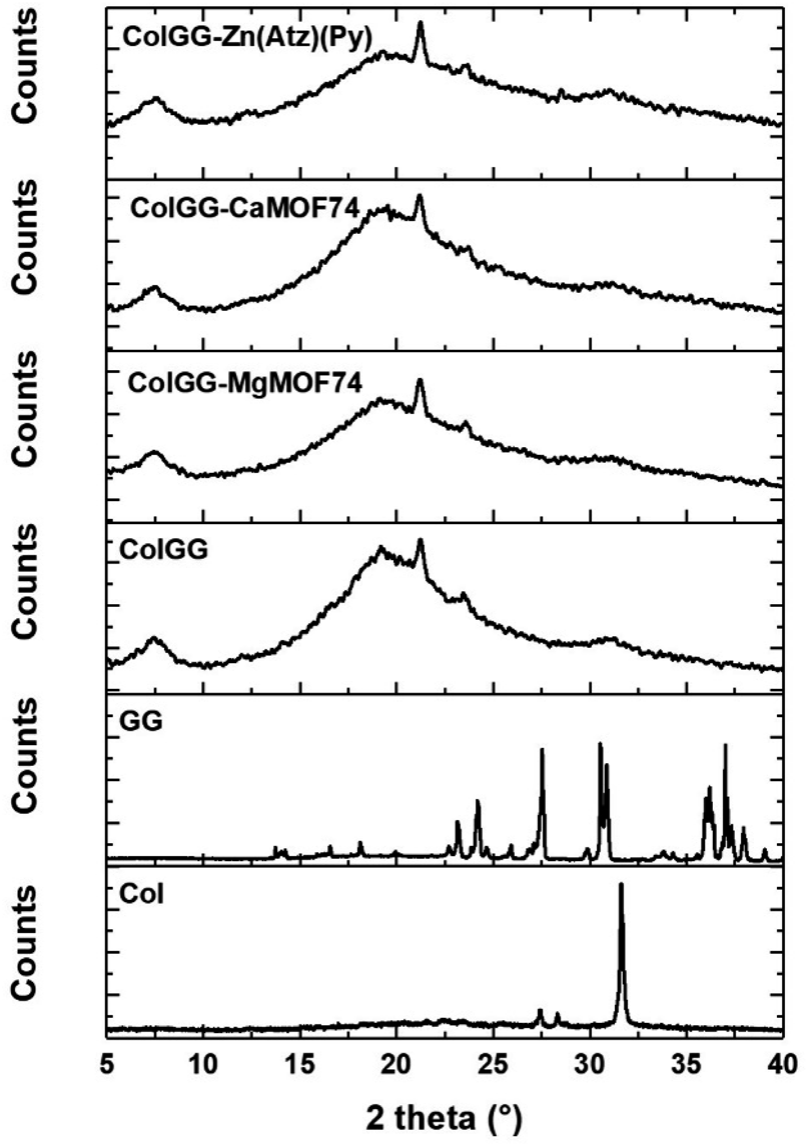
XRD patterns of composite materials.

The crystallinity of composite hydrogels was analyzed by WAXS and the obtained patterns are shown in Fig. 4. They revealed the amorphous nature of the ColGG matrix and composites. The pattern of ColGG shows broad halos between 5 and 40° and a weak diffraction peak at 2*θ* = 21.4° and thus, the polymer matrix is predominantly amorphous. This peak is absent in the XRD pattern of collagen and guar gum and thus, results from the semi-interpenetration of collagen with guar gum. The XRD pattern of guar gum shows several diffraction peaks between 13 and 26°. While the XRD pattern of collagen only exhibits a strong diffraction peak at 32° which is attributed to the triple helix structure formed by the polypeptide chains. Therefore, the process of semi-interpenetration negatively affects the crystallinity of guar gum and collagen. Particularly, the peak at 21.4° suggests that the semi-interpenetration process disorders the structure of guar gum forming large amorphous regions in the polymer matrix. Reciprocally, guar gum disorder the triple helix of collagen leading to the broadening of the diffraction peak at 32°. The patterns of composites show the same characteristics of the pattern of ColGG showing that MOFs do not improve the crystallinity of semi-interpenetrating networks. Conversely, the amorphous character of the polymer matrix increases after adding MOFs. This increment in amorphous structure is associated with MOFs preventing polymeric packing and alignment during the formation of semi-IPN matrix, generating disordered regions that do not have the ability to diffract X radiation. The intermolecular hydrogen bonds generated by the MOF binders with the polymeric matrix prevent polymeric ordering. The presence of amorphous regions in wound healing scaffolds is required to guarantee cell adherence and growth.^44^

### Microscopy analysis

**Fig. 5:**
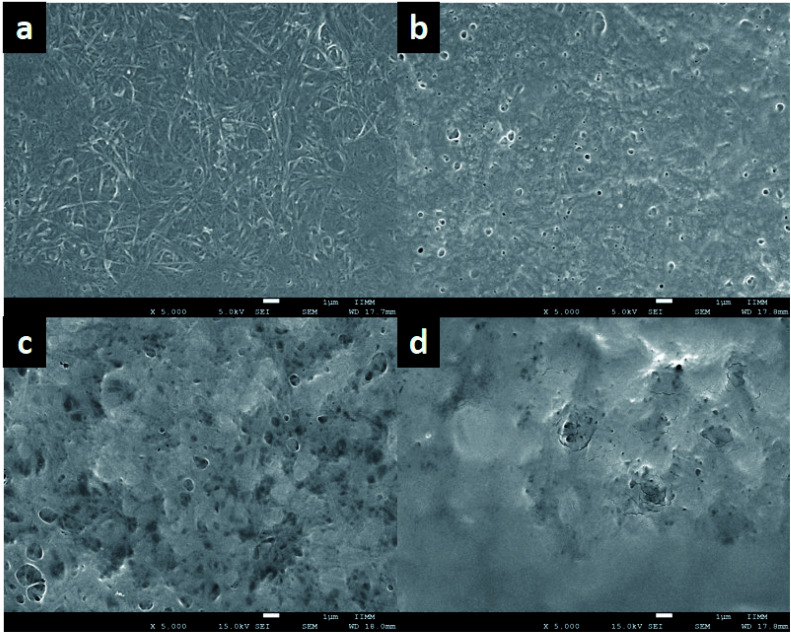
SEM images of (a) ColGG, (b) ColGG MgMOF74, (c) ColGG CaMOF74, and (d) ColGG Zn(Atz)(Py).

Fig. 5 show the SEM images of ColGG and composites containing MOFs. The micrograph of ColGG reveals an amorphous fibrillary surface with interconnected porosity (typical for collagen), also flat regions are embedded in this matrix (semi-interpenetration of the guar gum polysaccharide) (Fig. 5a). Micrographs of composites indicate that MOFs promote the nucleation of collagen fibrils by hydrogen bridge interactions, promoting amorphous granular regions with characteristic porosity, modifying the fibrillary nature.

**Fig. 6:**
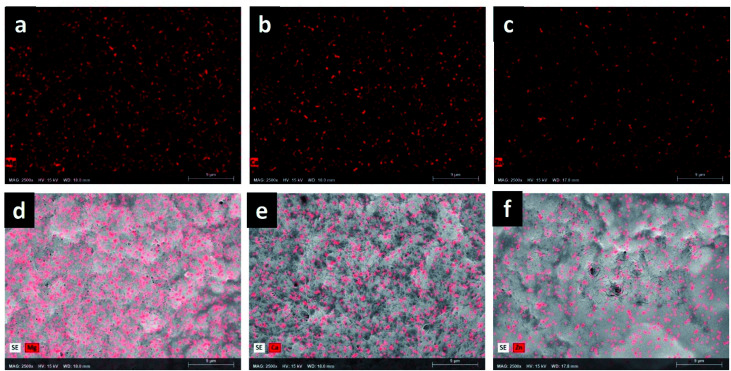
Elemental mapping acquired by EDS: (a) ColGG MgMOF74, (b) ColGG CaMOF74, (c) ColGG Zn(Atz)(Py); and elemental mapping acquired by secondary electrons (d) ColGG-MgMOF74, (e) ColGG-CaMOF74, (f) ColGG-Zn(Atz)(Py).

The variation in the gelation rate (*S*) and the storage modulus (*G*′) of the composites is associated with this structural modification that each MOF produces in the biopolymeric matrix. These results confirm the observations realized by WAXS. To obtain more information about the dispersion and distribution of MOFs in composites, elemental mapping was performed by EDX. Fig. 6a–c reveal that elements Mg, Ca, and Zn are uniformly distributed in the polymeric matrix, which suggests a uniform distribution of MOFs in the composite. These observations were confirmed in the images acquired with secondary electrons (Fig. 6d–f).

Surface porosity is a fundamental characteristic to allow basic cellular functions such as migration, growth, and proliferation, which is essential for tissue healing.^45^ Likewise, the homogeneous distribution of MOFs in the biopolymeric matrix allows guaranteeing their possible biological functionality, such as controlled release of drugs, antimicrobial capacity, and modulation of the metabolism of inflammation, angiogenesis or fibrogenesis.

### Swelling and degradation behavior

**Fig. 7:**
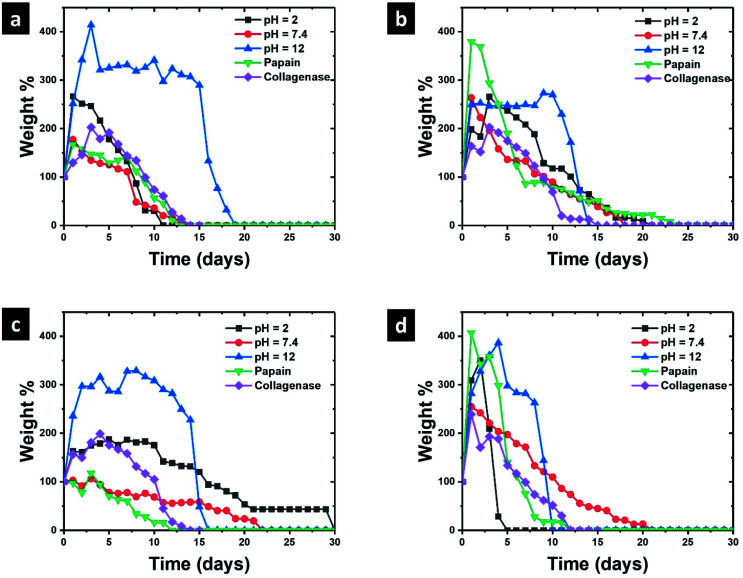
Degradation profiles for (a) ColGG, (b) ColGG-MgMOF74, (c) ColGG-CaMOF74, and (d) ColGG-Zn(Atz)(Py).

Fig. 7 presents the swelling/degradation profiles in hydrolytic and proteolytic media of ColGG and composites reinforced with MOFs. The xerogel of ColGG shows a high water uptake capacity in the alkaline medium reaching a swelling degree of 415 ± 40% after 3 days of contact (Fig. 7a). After that, the swelling of the hydrogel decreased to 300 ± 30% and remained without significant variation for 9 days. Then, the hydrogel lost mass continuously due to hydrolytic degradation. Finally, the hydrogel ColGG decomposes completely after 19 days. ColGG exhibits similar swelling/degradation profiles in proteolytic media (papain and collagenase) and pH 7.4 reaching a maximum swelling of 200 ± 25 and being completely degraded after 14 days.

The incorporation of MOFs affects the swelling/degradation behavior of the ColGG matrix. MgMOF74, CaMOF74, and Zn(Atz)(Py) decrease the swelling capacity of the ColGG matrix and reduced the required time for degradation in the alkaline medium. The amorphous granular regions of collagen–guar gum promoted by MOFs show susceptibility to alkaline degradation, limiting the swelling capacity of the composite matrix. Conversely, MOFs improved the stability of ColGG xerogels at pH 2 and 7.4 slowing the degradation rate of hydrogels. The hydrogen bond interactions of the MOF binders with the biopolymer matrix enhance the resistance to both acidic and neutral degradation. Finally, MOFs do not have a significant effect on the swelling/degradation behavior of ColGG and composites in the proteolytic medium. The resistance to degradation that MOFs provide to the composite matrix could be used to promote controlled release of drugs such as antibiotics, anti-inflammatories, or molecules of interest in wound healing. While the modulation of the swelling of the matrix in hydrolytic media can be used for the absorption of wound exudates, promoting healing, and preventing the spread of infections.^46^

### Evaluation of the *in vitro* biocompatibility

#### Cell viability

**Fig. 8:**
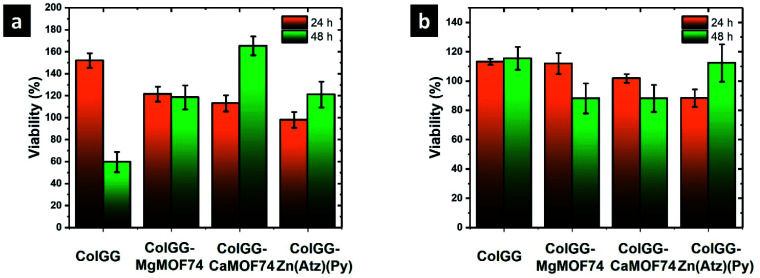
Cell viability of (a) porcine fibroblasts, and (b) human monocytes.

The biocompatibility of ColGG and the composites was evaluated measuring the cell viability by the MTT assay using fibroblasts from the porcine dermis and human monocytes (Fig. 8). In this assay, 60% of cell viability is considered as the lowest permissible value to consider the material as non-cytotoxic.^47^ The viability of fibroblasts in contact with the ColGG hydrogel is 157 ± 7% at 24 h of contact. Composites containing MOFs show lower cell viability than ColGG at the same time of evaluation. The cell viability for composites containing MgMOF74, CaMOF74, and Zn(Atz)(Py) is 121 ± 7, 113 ± 7, and 97 ± 7%, respectively. However, at 48 h the viability of fibroblasts in contact with ColGG decreases to 59 ± 9%, while the composites ColGG-CaMOF74 and ColGG-Zn(Atz)(Py) increase to 165 ± 8 and 121 ± 12%, respectively. The viability of monocytes at 24 h is 113 ± 2, 112 ± 7, 102 ± 3, and 88 ± 6 for ColGG, ColGG-MgMOF74, ColGG-CaMOF74, and ColGG-Zn(Atz)(Py), respectively. At 48 h, the viability of monocytes in contact with ColGG remains without significant differences, while the viability on ColGG-CaMOF74 and ColGG-MgMOF74 is lower than 90%. Finally, the viability of monocytes on ColGG-Zn(Atz)(Py) increases up to 112 ± 13% at 48 h.

Results showed that the formulated hydrogels are not cytotoxic. The chemical composition of the composite scaffolds does not alter the breathing capacity of both monocytes and fibroblasts growing on these structures, indicating that cells important in the healing process have an active metabolism. Monocytes regulate inflammation processes and provide protection against infection, and fibroblasts help in the construction of new tissue.^48^ Specifically, CaMOF74 promotes the metabolic activity of monocytes, ensuring their viability up to 48 h, and Zn(Atz)(Py) stimulates the metabolism of fibroblasts. Further, the hemolysis assay showed 0% of hemolysis for all the tested materials (data not shown). The high biocompatibility of composite scaffolds is due to the composition of the polymeric matrix formed by collagen and guar gum; the products of the degradation of these scaffolds such as amino acids, galactose, mannose, and glycerol stimulate cell viability and do not show hemolytic character. Therefore, the incorporation of MOFs such as MgMOF74, CaMOF74, and Zn(Atz)(Py) could tailor the biocompatibility of hydrogels. MOFs may release Mg^2+^, Ca^2+^, and Zn^2+^ ions which are useful for several cell functions. Mg^2+^ and Ca^2+^ are two of the more abundant ions in the tissues and biological fluids of mammals. They have structural functions such as the stabilization of the cell membrane, important enzyme control and antimicrobial capacity.^49^ While Zn^2+^ occurs in several fundamental metalloenzymes which participate in the constitution and degradation of proteins, lipids, and nucleic acids, in the stabilization of protein structures, in control and regulation processes, and the transfer of genetic information.^50^ Thus, the continuous releases of these ions could tailor the cell function improving the performance of these composite scaffolds for the healing of chronic wounds.

#### Cell proliferation

**Fig. 9:**
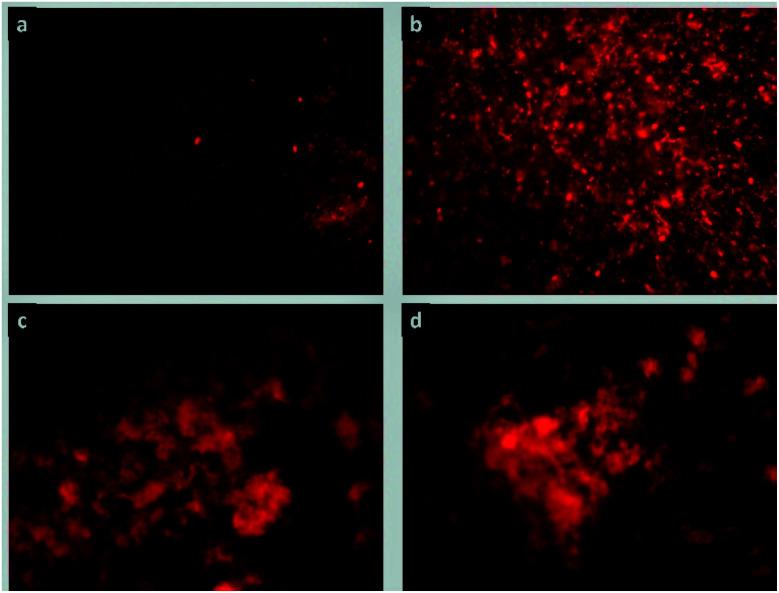
Proliferation images of porcine fibroblasts stained with rhodamine B in contact with hydrogel composites: (a) ColGG, (b) ColGG-MgMOF74, (c) ColGG-CaMOF74, and (d) Zn(Atz)(Py).

**Fig. 10:**
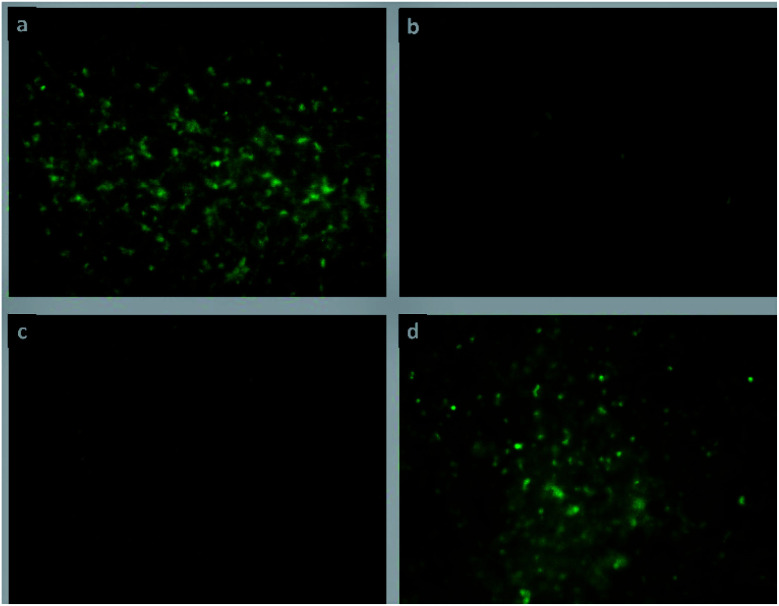
Proliferation images of porcine fibroblasts stained with calcein in contact with hydrogel composites: (a) ColGG, (b) ColGG-MgMOF74, (c) ColGG-CaMOF74, and (d) Zn(Atz)(Py).

Results obtained by the MTT assay were confirmed by inspecting the fibroblast proliferation by fluorescence microscopy staining. Fig. 9 presents images of fibroblasts stained with rhodamine B at an incubation time of 48 h. The images reveal dense populations of living fibroblasts in contact with the matrix ColGG and composites containing MOFs. Fig. 10 shows images of fibroblasts stained with calcein confirming that fibroblasts conserve their active breathing capacity in contact with the degradation by-products of ColGG and their composites and thus, they can deacetylate the calcein acetoxymethyl ester yielding the fluorescent green calcein label.^51^Fig. 10a and d show dense fibroblast populations compared with Fig. 10b and c showing that ColGG and ColGG-Zn(Atz)(Py) stimulate better proliferation than composites containing MgMOF74 and CaMOF74. Results obtained by fluorescence microscopy and the MTT assay confirmed that ColGG and the composites containing MOFs allow the growth and proliferation of cells involved in tissue regeneration. Cell proliferation is a basic function of biological systems, where cells grow, migrate, and interrelate to generate tissues with specific functions. Ensuring that the composition of the scaffolds in the hydrogel state allows cell proliferation could be associated with success in application as dressings for wound healing. The agglomerated morphology of granular regions that Zn(Atz)(Py) MOF generates, allows fibroblasts to proliferate abundantly on this matrix. The flat regions with higher physicochemical crosslinking promoted by the hydrogen bonds that the carboxylate binders of the CaMOF74 and MgMOF74 have with the biopolymeric matrix present less release of degradation by-products, limiting cell proliferation.

#### Cell signaling

**Fig. 11:**
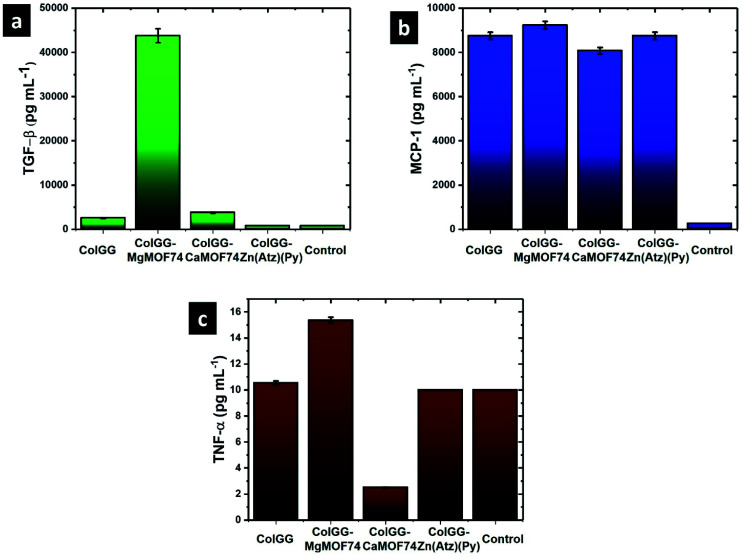
Concentration of cytokines secreted by human monocytes after being in contact with composite hydrogels for 48 h: (a) TGF-β, (b) MCP-1, and (c) TNF-α.

To evaluate how the composition of the composite hydrogels affects the biologic response during the tissue regeneration, it was studied the secretion of certain growth factors and cytokines in such as TGF-β, MCP-1, and TNF-α related with the construction of new tissue, modulation of the inflammatory response and cell lysis, respectively (Fig. 11).^52^ After incubating human monocytes in contact with the composite hydrogels the secretion of TGF-β is stimulated strongly by ColGG-MgMOF74 reaching a concentration of 43 750 ± 1588 pg mL^−1^. Also, the production of this cytokine is stimulated by ColGG and the composite containing CaMOF74. Conversely, the composite ColGG-Zn(Atz)(Py) does not make changes in the production of TGF-β founding the typical concentration observed in human blood (700 pg mL^−1^). The degradation products and their associations with Mg^2+^ ions and the ColGG-MgMOF74 structure make it possible to adapt monocyte signaling for higher TGF-β segregation, indicating that this composite scaffold can be successfully used for tissue regeneration.

The protein MCP-1 is responsible for recruiting monocytes, T cells, and dendritic cells to the site of inflammation caused by tissue injury. All the evaluated materials stimulated the production of this important protein, the maximum concentration of MCP-1 was observed with ColGG-MgMOF74 (9231 ± 173 pg mL^−1^). For all the evaluated materials, the concentration of MCP-1 is significantly higher than the typical values for blood (2500 pg mL^−1^). Thus, it was confirmed that the evaluated composite hydrogels containing MOFs stimulate monocyte signaling to secrete a higher content of MCP-1, and the metal ion of the MOF does not influence on this stimulation, indicating that this improvement is associated with the composition of the biopolymeric matrix. Collagen and guar gum allow monocytes to have an active metabolism that promotes the control of inflammation (high secretion of MCP-1).

Finally, ColGG, ColGG-MgMOF74, and ColGG-Zn(Atz)(Py) do not produce significant changes in the secretion of TNF-α compared with the typical values observed in the blood (10 pg mL^−1^). Conversely, it is observed that ColGG-CaMOF74 inhibited the secretion of TNF-α. High levels of this factor indicate processes of cell lysis, associated with the formation of necrotized tissues and/or associated with scar formation. The composite scaffold that has MOF containing the Ca^2+^ ion of higher atomic size, allows that the signaling of the monocytes tends to the lower production of tumor necrosis factor, which it could be used to control inflammation avoiding the formation of necrotized or poorly healed tissues if this composite scaffold is used as wound dressing.

#### Antibacterial activity

**Fig. 12:**
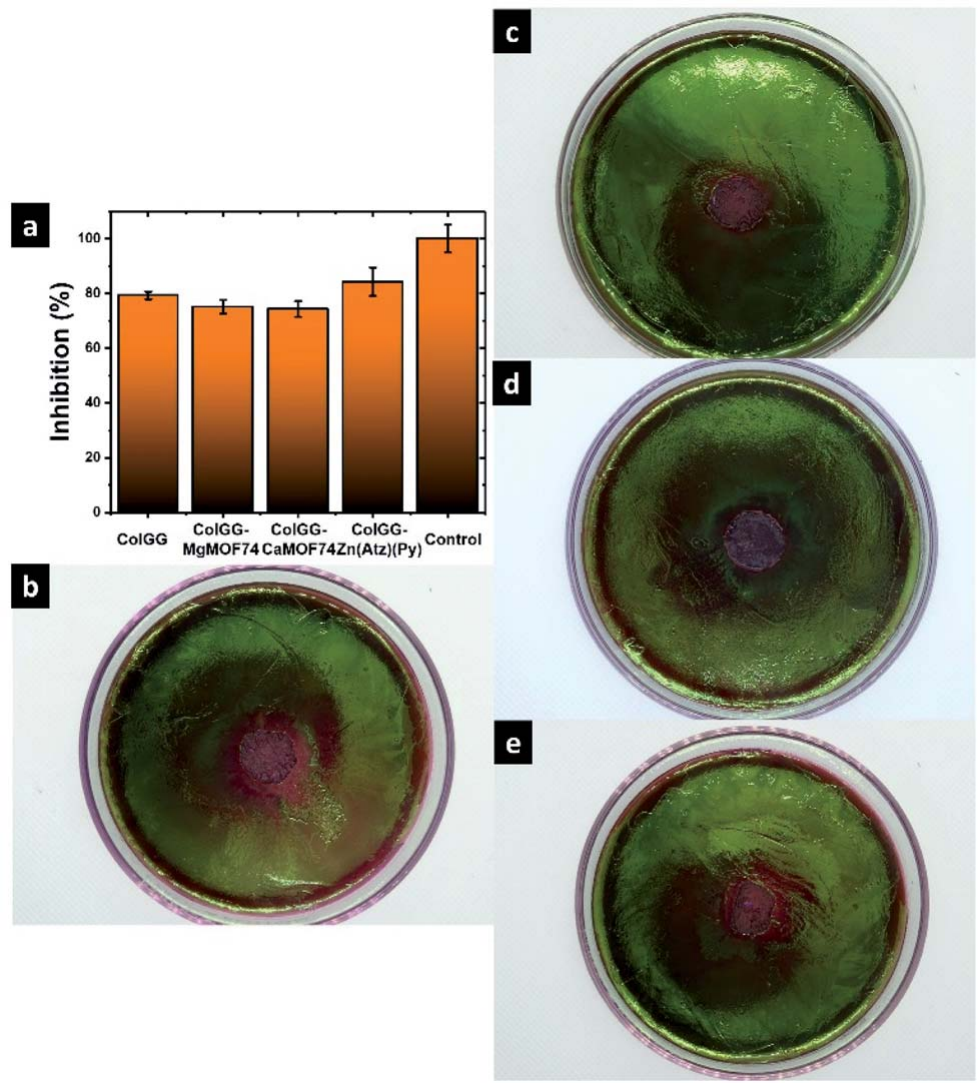
(a) Inhibition ratio of *E. coli* and antibiograms of (b) ColGG, (c) ColGG MgMOF74, (d) ColGG CaMOF74, and (e) ColGG Zn(Atz)(Py).

To avoid the growth of pathogen microorganisms is an important function of biomaterials for tissue engineering because infections difficult the correct wound healing. Fig. 12a shows the inhibition capacity of composite hydrogels against *Escherichia coli* and Table 3 shows the data from the inhibition assays. The ColGG hydrogel exhibits an inhibition capacity of 79.2 ± 1.44%. The addition of MgMOF74 and CaMOF74 does not improve the inhibition capacity showing lower inhibition than ColGG. However, the incorporation of Zn(Atz)(Py) increases the inhibition capacity up to 84.2 ± 5.2%. Finally, Fig. 12b–e shows the antibiograms of ColGG, ColGG-MgMOF74, ColGG-CaMOF74, ColGG-Zn(Atz)(Py), respectively.

**Table 3:** Data from bacterial inhibition

	Inhibition halo (cm)	Inhibition capacity of *E. Coli* (%)	Standard deviation (cm, %)
ColGG	1.58	79.2	0.03, 1.44
ColGG-MgMOF74	1.50	75	0.05, 2.50
ColdsluG-CaMOF74	1.48	74.2	0.06, 2.89
ColGG-Zn(Atz)(Py)	1.68	84.2	0.10, 5.20
Control	2	100	0.05, 2.50

Previous works reported that yttrium(iii) complexes of 2,6-pyridinedicarboxylic acid have low antibacterial activity against *E. coli* and *S. aureus*. These complexes interfere with the transport of substrates and ions through the cell membrane. However, their low inhibition capacity was attributed to the low inhibition capacity of the yttrium ion.^53,54^ In other research, Soltani *et al.* reported that nickel(ii) complexes of 2,6-pyridinedicarboxylic acid have lower inhibition capacity than their constituents evaluated individually. Nonetheless, they conclude that complexes are better antibacterial agents because they release progressively the ions and ligands with higher antibacterial activity.^55^ Conversely, copper(ii) complexes of 2,6-pyridinedicarboxylate showed antibacterial activity comparable with common antimicrobials such as gentamycin and fluconazole.^56^ Thus, the inhibition capacity of metal complexes containing the ligand 2,6-pyridinedicarboxylic acid depends on the metal ion. For this, Zn(Atz)(Py) shows higher antibacterial activity than MgMOF74 and CaMOF74. Zn-MOFs based on the ligand 2,6-pyridinedicarboxylic acid have presented high antibacterial activity previously.^57^ For this, Zn^2+^ could enhance the antibacterial properties of Zn(Atz)(Py). This MOF contains the 3-amino-1,2,4-triazole ligand, which is a compound with a broad spectrum of biological activities, including antibacterial, antifungal, and antiviral. The organic ligands containing the 1,2,4-triazole moiety show high inhibition capacity against several microorganisms, while their metal complexes showed varying degrees of inhibition.^58^ From the above, 3-amino-1,2,4-triazole may contribute to improving the antibacterial performance of Zn(Atz)(Py). This ability to inhibit the growth of pathogens such as *E. coli* that the ColGG-Zn(Atz)(Py) composite presents could be used to function as a dressing for the healing of chronic wounds, since chronic wounds have difficulty in healing due to the presence of this type of microorganisms.

#### Release profiles of ketorolac

**Fig. 13:**
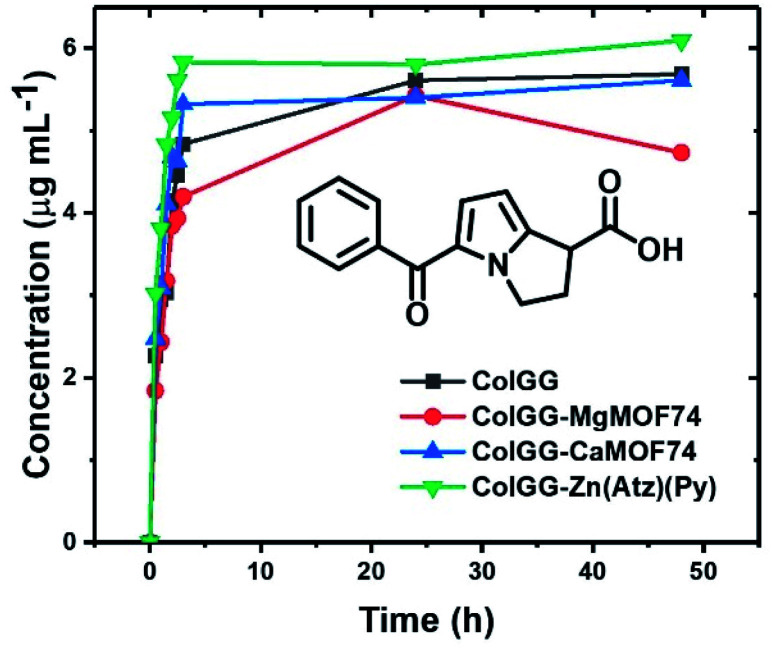
Release profiles of ketorolac in PBS 1X at 37 °C.

Another important function of hydrogels for wound healing therapy is the controlled release of substances with therapeutic function. Ketorolac is a non-steroidal anti-inflammatory widely used for wound healing.^59^Fig. 13 shows the release profiles of ketorolac in PBS-1X (pH 7.4) at 37 °C. The release profiles reveal that the concentration of ketorolac in the medium increases rapidly during the first 3 h of contact. After that time, the concentration of ketorolac does not increase significantly. The maximum values of concentration of released ketorolac were 5.6 ± 0.5, 4.7 ± 0.4, 5.6 ± 0.5, and 6.1 ± 0.5 μg mL^−1^ for ColGG, and the composites containing MgMOF74, CaMOF74, and Zn(Atz)(Py), respectively. These values represent a released mass of drug of 112 ± 10, 94 ± 8, 112 ± 10, and 122 ± 10 μg. Thus, the release efficiency of ketorolac was 28 ± 2.5, 23.5 ± 2, 28 ± 2.5, and 30.5 ± 2.5% for ColGG, ColGG-MgMOF74, ColGG-CaMOF74, and ColGG-Zn(Atz)(Py), respectively, with respect to the initial mass of loaded ketorolac in the hydrogels. These results indicate that ketorolac is strongly retained in the polymer matrix by intermolecular forces, mainly hydrogen bonding, limiting your release under these conditions. According to the evaluated structural and physicochemical properties of composite hydrogels, the surfaces with higher granular agglomeration based on Zn(Atz)(Py) allow higher release of the drug of interest. These results could be used to load inside composites other molecules with therapeutic interest in wound healing, such as anti-inflammatories, analgesics, and/or antibiotics, where the controlled release promoted by the structure of the composite scaffolds could benefit the healing process if they are used as dressings.

## Experimental

### Materials

Collagen from porcine dermis was extracted by enzymatic hydrolysis following the procedure reported elsewhere.^60^ A polyurethane prepolymer used as the crosslinking agent was synthesized reacting hexamethylene diisocyanate with glycerol ethoxylate as reported in.^61^ Guar gum (extracted from *Cyamopsis tetragonoloba*, *M*_w_ ≈ 220 kDa) and the reagents used for the synthesis of MOFs and polyurethane are commercially available in Sigma Aldrich Co.

### Synthesis of MOFs

CaMOF74 and MgMOF74 were synthesized under hydrothermal conditions based on the methodology reported by Ranjbar.^62^ For this, 2.9307 g (17.4 mmol) of 2,6-pyridinedicarboxylic acid (H_2_Py) and 1.4030 g (34.8 mmol) of NaOH were dissolved in 30 mL of water. Then, 8.2825 g (34.8 mmol) of Ca(NO_3_)_2_·4H_2_O or 8.9031 g (34.8 mmol) Mg(NO_3_)_2_·6H_2_O previously dissolved in 20 mL of water were added. The mixture was vigorously stirred for 5 min and transferred to a teflon-lined hydrothermal autoclave and heated at 120 °C for 72 h. The solid product was filtered, rinsed with water, and dried at 70 °C for 24 h. CaMOF74, yield: 3.0508 g (21.9% respect to Ca). MgMOF74, yield: 4.0294 g (31.7% respect to Mg).

Zn(Py)(Atz) was synthesized under hydrothermal conditions based on the procedure reported by Lan and co-workers.^63^ We prepared three solutions with the precursors. Solution 1 was prepared dissolving 2.9307 g (0.0174 mmol) of 2,6-pyridinedicarboxylic acid and 1.40230 g (0.0348 mmol) of NaOH in 20 mL of water. The solution 2 was prepared dissolving 1.5392 g (0.0174 mmol) of 3-amino-1,2,4-triazole (HAtz) in 20 mL of water. The solution 3 was prepared dissolving 5.2819 g (0.0174 mmol) of Zn(NO_3_)_2_·6H_2_O in 10 mL of water. Solutions 1 and 2 were mixed under vigorous stirring for 5 min. Then, solution 3 was added under continuous stirring and transferred to a teflon-lined hydrothermal autoclave and heated at 100 °C for 72 h. The solid product was filtered, rinsed with water, and dried at 70 °C. Yield: 4.2303 g (49.7% respect to Zn).

### Preparation of composite hydrogels

The synthesis of the composite hydrogels was previously reported in.^41^ For the synthesis of semi-IPN hydrogels, a collagen solution was prepared (6 mg mL^−1^). For the synthesis of composites containing MgMOF74, CaMOF74, and Zn(Atz)(Py), a suspension of MOFs was prepared in the collagen solution (1% wt respect to collagen). 1 mL of collagen or MOF suspension was mixed with the polyurethane crosslinker (15% wt respect to collagen) at 4–5 °C. After that, guar gum was added (10% wt respect to collagen) and then, the pH of the mixture was adjusted at pH 7.4 by adding 300 μL of PBS-10X. Finally, the mixture was heated at 37 °C for 4 h. Table 4 presents the mass of collagen, polyurethane, guar gum and MOFs employed for the formulation of hydrogels.

**Table 4:** Designation and composition of composite hydrogels

Material	MOF (μg)	Collagen (mg)	Polyurethane (mg)	Guar gum (mg)
ColGG	0	6	0.9	0.06
ColGG-MgMOF74	60	6	0.9	0.06
ColGG-CaMOF74	60	6	0.9	0.06
ColGG-Zn(Atz)(Py)	60	6	0.9	0.06

### Characterization of composite hydrogels

FTIR spectra were recorded with a PerkinElmer Frontier spectrophotometer using an attenuated total reflection (ATR) accessory. Hydrogels were dehydrated before analyzing. The crystallinity of the composite materials was studied by wide-angle X-ray scattering (WAXS), utilizing a SAXS-Emc2 Anton Paar diffractometer equipped with a Cu Kα X-ray source (*λ* = 1.54 Å). The morphology of composite materials was observed by scanning electron microscopy using a JEOL JSM-6510LV/LGS microscope operated at 15 kV. Samples were covered with graphite to avoid the accumulation of electrostatic charge. The storage module (*G*′) of hydrogels were measured by low amplitude oscillatory rheometry using an Anton-Paar Physica MCR 301 rheometer. Experiments were carried out at 37 °C with a plate–plate geometry (diameter = 40 mm). Measurements were performed with a 10% of strain to ensure the viscoelastic behavior in the dynamic response of hydrogels.

### Gelling kinetics

The gelling kinetics for the composite matrix formation process was studied by turbidimetric analysis. Samples of 200 μL from the polymerizable mixtures were placed in a microplate and the absorbance at 406 nm was measured at 37 °C each 30 s for 90 min with a ThermoScientific MultiskanSky spectrophotometer. The gelation parameters such as nucleation time (*t*_lag_) (region with low absorbance values), average gelation time (*t*_1/2_) (time where half of the maximum absorbance value is reached) and gelation rate (*S*) (slope of the linear region of the curve) were obtained.

### Swelling/degradation behavior

The swelling and degradation behavior of xerogels was studied in hydrolytic and proteolytic media. The hydrolytic media were aqueous solutions with pH 2, 7.4, and 12. The proteolytic media were aqueous solutions of papain (14 U per gel) and collagenase (14 U per gel). The experiments were performed in closed jars placing one xerogel and 10 mL of the hydrolytic or proteolytic solution. Samples remained in repose at room temperature (25 ± 1 °C) and the mass of hydrogels was monitored at different time intervals. Experiments were performed in triplicate.

### Evaluation of the *in vitro* biocompatibility

#### Evaluation of cell viability

The variation of metabolism of human monocytes or porcine dermal fibroblasts (got from primary culture) growing on every composite hydrogel was assessed. For the extraction of fibroblasts from porcine skin, a piece of dermis was obtained at a local slaughterhouse. Monocytes were extracted from a human blood sample obtained from a healthy volunteer in the university clinical analysis laboratory. Cell suspensions in DMEM (for fibroblasts) or RPMI (for monocytes) culture medium were added to the wells containing hydrogels (50 000 cells per well) and control (without hydrogels), and these were incubated for 24 and 48 h, respectively. The cell viability was dictated by the limit of cells with dynamic metabolism to transform MTT salts in formazan. The MTT (1 wt/vol%) was added to wells with hydrogels and controls, and afterward, the cells were kept up under culture conditions for 3 h at 37 °C. At that point, the medium was tapped, the formazan was diluted in propan-2-ol, and the absorbance of the solutions was measured at 560 nm, using a ThermoScientific MultiSkanSky spectrophotometer. Cell viability was calculated with eqn (1):1
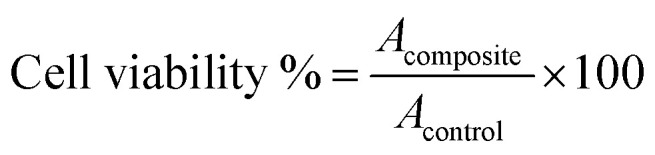
where *A*_composite_ and *A*_control_ are the absorbances for composite hydrogels and control (PBS-1X) respectively.

#### Cell proliferation

The growing of porcine fibroblasts in contact with the composite hydrogels for 48 h was evaluated with the live/dead fluorescence assay. Similarly, fibroblasts under the same conditions were stained with rhodamine B. Cell populations and proliferation were observed using a VELAB VE-146YT epifluorescence microscope.

#### Hemolysis assay

The hemocompatibility of hydrogels was evaluated by the hemolysis assay measuring the released hemoglobin after destroying the cell membrane of erythrocytes. For this, a human blood sample was obtained from a healthy volunteer in the university clinical analysis laboratory. Samples of erythrocytes previously purified in Alsever's solution (112 μL) were mixed with 150 μL of leached extracted from composite hydrogels, and 1728 μL of Alsever's solution. Alsever's solution, and deionized water were used as the negative (0% of hemolysis), and positive (100% of hemolysis) controls, respectively. Samples were incubated at 37 °C with orbital stirring (250 rpm) for 30 min. After that, samples were centrifuged at 3000 rpm and aliquots were taken from the supernatant. The absorbance of samples was measured at 415 nm with a ThermoScientific MultiskanSky spectrophotometer, and the percentage of hemolysis was calculated with eqn (2):2
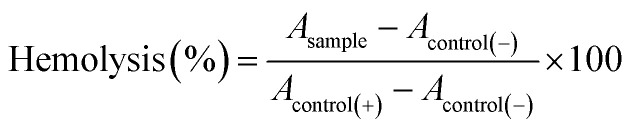
Where *A*_sample_, *A*_control(−)_, and *A*_control(+)_ are the absorbance of samples, negative control, and positive control, respectively.

#### Enzyme-linked immunosorbent assays

The effect of the composition of the composite hydrogels on cell signaling was evaluated using ELISA for the detection of important cytokines in tissue inflammation and reconstruction processes, such as beta transforming growth factor (TGFβ), monocyte chemoattractant protein (MCP-1), and alpha tumor necrosis factor (TNFα). The cytokines were determined in human monocytes growing in the presence of the composite materials for 48 h following the supplier's instructions (invitrogen). Cells growing in PBS-1X were used as a reference control.

#### Bacterial inhibition

*In vitro* bacterial inhibition tests were performed using eosin-methylene blue (EMB) agar and a bacterial strain of *E. coli* (lyophilized cells purchased from Sigma Aldrich Co.). The agar was prepared by dissolving 37.5 g of dehydrated agar in 1 L of distilled water and was heated until dissolved. The culture medium was sterilized between 121–124 °C for 15 minutes. Once the medium was ready, it was poured into Petri dishes which were previously sterilized. Dry composite hydrogels were placed in the center of the culture medium dishes, with the finality to measure the inhibition halo. Amoxicillin prepared at 20 ppm was used as a control. The bacterial strain was seeded homogeneously (1.5 × 10^8^ cell per ml) in the Petri dishes including hydrogels and control using a sterile loop. The bacterium was incubated for 24 h at 37 °C to measure its growth in the presence of the materials. The inhibition capacity of *E. coli* was calculated by comparing the diameter of the halo formed by the composite hydrogel with respect to the diameter generated by the amoxicillin.

#### Ketorolac release

The capability of composite hydrogels to release ketorolac was studied by release profiles. 400 μg of ketorolac were encapsulated in each type of composite hydrogel. Then, hydrogels were placed in PBS-1X at 37 °C (20 mL per hydrogel). Aliquots of the medium were taken at different time intervals and the concentration of ketorolac were measured at 325 nm with a ThermoScientific MultiSkanSky spectrophotometer.

## Conclusions

Composite hydrogels containing MOFs based on Mg, Ca, and Zn have a high potential to be used as biomaterials. MgMOF74, CaMOF74, and Zn(Atz)(Py) were incorporated in hydrogel matrixes based on collagen and guar gum. The bio-based hydrogel matrixes exhibit good biocompatibility which can be tailored for wound healing applications by adding the aforementioned MOFs. The best scaffold for this purpose was the ColGG-Zn(Atz)(Py) composite which presented higher viability for human monocytes and porcine fibroblasts. This material has no hemolytic character and tailored a required cell signaling in healing, increasing the secretion of the cytokine MCP-1 and maintaining the levels of TGF-β and TNF-α in normal values. The high biocompatibility of the ColGG-Zn(Atz)(Py) composite was confirmed by the proliferation images of porcine fibroblasts stained with calcein, showing that cells in contact with the composite can grow and proliferate forming large and dense populations. Finally, this composite showed high antibacterial inhibition against *E. coli* and exhibited the ability to release drugs such as ketorolac in a controlled way. From the above, the composite ColGG-Zn(Atz)(Py) has great potential for biomedical applications such as dressing for wound healing.

## Author contributions

M. C.-V.: investigation, methodology formal analysis, writing original draft, writing review and editing. D. A. C.-M.: validation, writing review and editing. J. J. B.-R.: investigation, validation, writing review and editing. J. A. C.-R.: methodology, conceptualization, funding acquisition, project administration, resources, supervision, review and editing.

## Conflicts of interest

There are no conflicts to declare.

## Supplementary Material
